# Development, Stability, and In Vitro/In Vivo Studies of Volatile Oil Pickering Emulsion Stabilized by Modified Amber

**DOI:** 10.3390/ph17091117

**Published:** 2024-08-24

**Authors:** Maomao Zhu, Zhonghuan Qu, Yanjun Yang, Ruyu Shi, Bing Yang, Yajun Shi, Junbo Zou, Xiaobin Jia

**Affiliations:** 1 Innovation Center for Industry-Education Integration of Pediatrics and Traditional Chinese Medicine, State Key Laboratory of Natural Medicines, School of Traditional Chinese Pharmacy, China Pharmaceutical University, Nanjing 211198, China15706037600@163.com (Y.Y.); s951001@126.com (R.S.); 15751151582@163.com (B.Y.); 2 Shaanxi Province Key Laboratory of New Drugs and Chinese Medicine Foundation Research, College of Pharmacy, Shaanxi University of Chinese Medicine, Xianyang 712046, China2051004@sntcm.edu.cn (Y.S.)

**Keywords:** *Acorus tatarinowii* volatile oil, stability, Pickering emulsion, modified medicinal particles, pharmacokinetics

## Abstract

Volatile oil stabilization strategies based on encapsulation with a large number of excipients limit further applications. The primary objective of this study is to improve the stability of volatile oils using Pickering emulsion (PE) stabilized by Chinese medicinal powder based on the principle of “integrating drug and excipient”. Modified amber was acquired through surface modification, and a stable oil-in-water PE loaded with *Acorus tatarinowii* volatile oil (ATVO) was constructed from modified amber. The stability, including the peroxide value (PV), malondialdehyde (MDA) content, and the content and composition of volatile components in modified amber-PE (MAPE) under intense light exposure, was analyzed deeply. In addition, the in vitro release and pharmacokinetics of MAPE and ATVO were investigated. The results demonstrate that the PV and MDA content in MAPE were significantly lower than in free ATVO, and the content and composition of volatile components in MAPE were closer to those in untreated ATVO. The release kinetics of *β*-asarone and *α*-asarone in MAPE demonstrated rapid and higher release, and pharmacokinetic studies show that MAPE has better bioavailability. This research provides a distinctive Chinese medicine solution to address the vaporization of volatile oil in solid formulations.

## 1. Introduction

Volatile oils (VOs) are major bioactive natural compounds [1] that have garnered considerable attention for their widespread pharmacological activities. Traditional Chinese medicine indicates that VOs have effects such as dispersing external pathogens, resolving dampness, regulating Qi circulation, and inducing resuscitation [2]. Modern pharmacological research has demonstrated that VOs possess properties such as anti-inflammatory, antioxidant, antimicrobial, anxiolytic, and wound healing effects [3,4]. Nonetheless, VOs are prone to volatilization, decomposition, and oxidation under conditions of light, heat, and oxygen exposure [5,6]. The current stabilization strategy for volatile oils in Chinese medicine mainly relies on the use of a large amount of excipients for “encapsulation” or “absorption”, which has improved the stability of the VOs but brought new problems. Cyclodextrins are widely used in the pharmaceutical field due to their clear composition, fewer adverse reactions, good safety, and high biocompatibility [7,8]. Furthermore, the cyclodextrin encapsulation technique has been reported to be utilized for the purpose of improving the stability of oils such as cinnamon oil [9], *Illicium verum* essential oil [10], and *Curcuma longa* volatile oil [11]. However, the cyclodextrin encapsulation technique has certain requirements concerning the physicochemical properties and molecular size of the volatile oils and relies on a higher proportion of cyclodextrins [12,13].

Polymer-based delivery systems mainly encapsulate volatile oils via nanocapsules, nanomicelles, nanogels, etc., exhibiting enhanced stability and water solubility [14]. However, the in vivo degradation of these polymers may result in toxicity, and residual organic solvents from the preparation process can also affect the safety of the medication [15]. Lipid-based delivery systems used to improve the stability of volatile oils have advantages such as enhancing the stability and solubility of volatile oils in water-soluble media, delaying the escape of volatile oils, avoiding oxidative degradation, and facilitating storage and use [13]. However, liposomes are sensitive to pH and temperature, and long-term storage may lead to problems such as phospholipid oxidation, leakage of essential oils, and particle aggregation [16]. The use of porous media adsorption and solidification technology to adsorb and solidify volatile oils provides a new method for improving the stability of volatile oils [17], but a large number of exogenous excipients are inevitably introduced [12]. Therefore, a stabilization strategy for VOs with minimal or no introduction of exogenous excipients, along with high safety and stability, is a pressing bottleneck issue.

Pickering emulsions (PE), stabilized by solid particles, are extensively utilized in the the food industry [18], cosmetics [19], and drug delivery [20] due to their excellent stability, good biocompatibility, and low toxicity [21]. The solid particles adsorbed at the oil-water interface form a closed-shell structure, which can effectively encapsulate the dispersed phase and prevent the occurrence of phase leakage or droplet rupture. Zhang et al. [22] demonstrated that the puerarin nanocrystalline self-stabilized Pickering emulsion showed long-term stability for up to 6 months without any phase separation or oil droplet coalescence. Additionally, PE has been shown to provide robust protection to VOs against oxidation [23] and can also enhance the dispersibility of VOs [24,25]. Lingzhu Pulvis is a typical Chinese medicine solid preparation that contains VOs, composed of *Acorus tatarinowii* volatile oil (ATVO), amber, pearl, and various other medicinal solid powders. However, the ATVO tends to volatilize easily, posing practical challenges to its stability in Lingzhu Pulvis [26]. Based on the concept of integrating drugs and excipients, this research team has proposed the idea of utilizing the medicinal solid powders in Chinese medicine prescriptions as stabilizers for preparing PE, aiming to improve the stability of VOs while minimizing the introduction of other excipients. This study aims to use Lingzhu Pulvis as a representative case to address this practical challenge.

In this study, modified amber with suitable wettability was obtained using PEG6000 (Tianjin Kemiou Chemical Reagent Co., Ltd., Tianjin, China) as the modifier through the melt method and was further used to stabilize an oil-in-water Pickering emulsion loaded with ATVO. Within this PE system, the stability of the loaded ATVO under a strong light environment was systematically studied. Furthermore, the in vitro release behavior and in vivo pharmacokinetics of both the loaded ATVO in the PE system and the free ATVO were investigated. The purpose of this work is to explore the possible effects of the Pickering emulsion system stabilized by modified Chinese medicinal solid powders, on the stability and behavior of volatile oils in both in vitro and in vivo settings, thus providing a research example for solid formulations of Chinese medicine containing VOs.

## 2. Results

### 2.1. Characterization of Modified Amber

**Figure 1:**
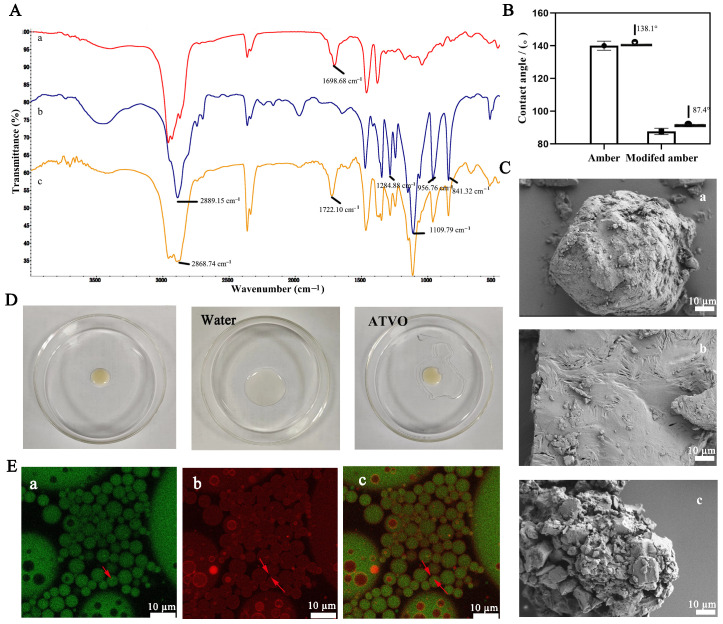
Characterization of modified amber and modified amber-PE (MAPE). (**A**) FTIR of amber (a), PEG6000 (b), and modified amber (c). (**B**) Contact angle of amber and modified amber. (**C**) SEM images of amber (a), PEG6000 (b), and modified amber (c). (**D**) Droplet diagram of MAPE. (**E**) CLSM images of MAPE (a Nile red, b Nile blue, c Merged).

The FTIR spectra of amber, PEG6000, and modified amber are presented in Figure 1A. The absorption peak of PEG6000 at 2889.15 cm^−1^ indicated the saturated C-H stretching vibrations, which shifted to 2868.74 cm^−1^ in modified amber. The characteristic peak of amber at 1698.68 cm^−1^ shifted to 1722.10 cm^−1^ after modification, which might be due to the stretching vibration peak of C=O. The FTIR of modified amber implied a corresponding absorption of PEG6000. The above results suggest that modified amber might share a similar chemical structure or composition with PEG6000, demonstrating a successful surface modification of amber particles.

The air-water contact angle was measured to assess the wettability of modified amber [27]. The contact angle of amber was 138.1° (Figure 1B), indicating its hydrophobic nature. After modification with PEG6000, there was a significant increase in the hydrophilicity of modified amber, with the contact angle decreasing from 138.1° to 87.4°. Generally, solid particles with a contact angle less than 90° are considered more suitable for stabilizing O/W-type emulsions [28]. The morphology of modified amber was investigated using scanning electron microscopy (Figure 1C). The amber particles exhibited a typical irregular block shape, and PEG6000 showed a neat block shape with a relatively smooth surface. Interestingly, the modified amber appeared as an aggregation of particles of varying sizes, evidencing the successful modification of its morphology by the melt method.

### 2.2. Characterization of MAPE

Both drop dilution and CLSM experiments indicate that the modified amber-PE (MAPE) was of the O/W type. The morphology of the MAPE droplet remained unchanged with the addition of ATVO droplets (Figure 1D), suggesting a non-oil-based continuous phase for MAPE. However, MAPE rapidly dispersed upon the addition of water droplets, demonstrating a water-based continuous phase. The CLSM experiment was used to further evaluate whether modified amber particles adsorb at the oil/water interface [29]. Figure 1E shows the CLSM images of MAPE, where the red region corresponds to modified amber particles stained with Nile Blue, whereas the green spots correspond to the ATVO phase stained with Nile Red. The MAPE system exhibited an oil-in-water configuration, with the oil droplets being efficiently dispersed in the aqueous phase (background color). In the merged CLSM image, the highlighted fluorescence rings (labeled in red) from modified amber mainly existed at the periphery of oil droplets, indicating that the modified amber particles were predominantly distributed on the surface of oil droplets. However, small emulsion droplets could be clearly observed within some large emulsion droplets. This may be related to the complex surface properties of modified amber particles, leading to the formation of multiple emulsions. The analysis performed with the Microtrac S3500 laser particle size analyzer showed that the droplet size of the MAPE system was 37.43 ± 1.36 µm.

### 2.3. Stability Analysis of ATVO in MAPE under Strong Lighting

#### 2.3.1. Oxidative Stability

**Figure 2:**
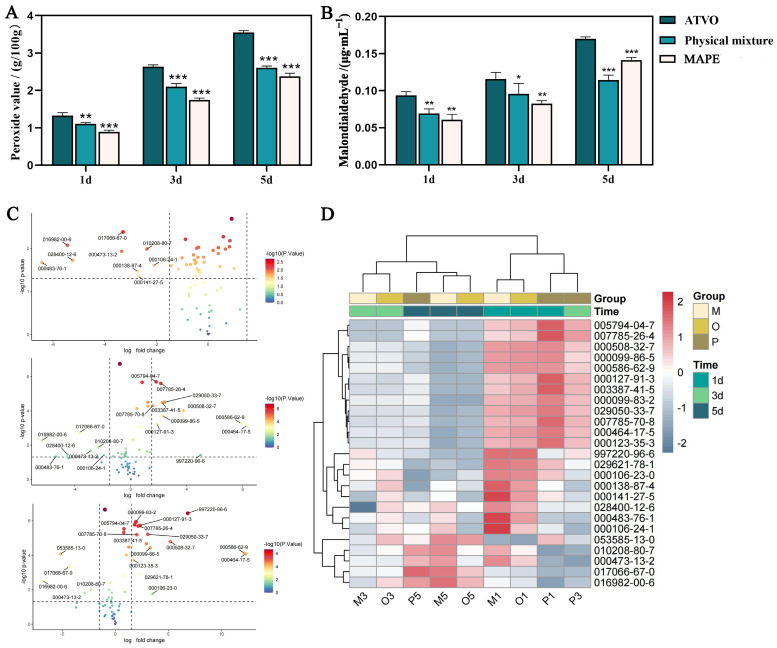
Results of the stability study. Peroxide value (**A**) and malondialdehyde content (**B**) of ATVO in MAPE, physical mixture, and ATVO under intense light exposure for 1, 3, and 5 d. (**C**) Volcano plot of volatile components between untreated ATVO and ATVO, which were processed for 1, 3, and 5 d under intense light exposure. (**D**) Heatmap of the average relative content of 25 differential volatile components in MAPE, physical mixture, and ATVO groups under intense light exposure. Data were $\bar{x}$ ± sd, n = 3; * *p <* 0.05, ** *p <* 0.01, and *** *p <* 0.001 vs. the ATVO group.

Light is a crucial factor affecting the stability of VOs through various pathways such as auto-oxidation [30] and photo-oxidation [31], leading to oxidative decomposition and isomerization of volatile components. Peroxide value (PV) is an important indicator for the initial stage of oxidation that reflects the levels of reactive oxygen and hydroperoxides [32]. The PV of ATVO in the three groups under a strong light environment is shown in Figure 2A. After 1, 3, and 5 d of intense light exposure, the PV in the ATVO group was recorded at 1.326, 2.634, and 3.551 g/100 g, respectively. Meanwhile, the PV of ATVO in the MAPE group was 0.888, 1.747, and 2.377 g/100 g at the corresponding time (*p* < 0.001).

Malondialdehyde is a type of aldehyde produced in large quantities during secondary lipid oxidation [33]. The MDA content in the ATVO group increased with prolonged exposure to strong light (Figure 2B). After treatment under an intense light environment for 1, 3, and 5 d, the MDA levels in the ATVO group were 0.094, 0.116, and 0.170 μg/mL, respectively. In contrast, the MDA levels in MAPE were 0.061, 0.082, and 0.141 μg/mL, demonstrating significantly lower levels compared to the ATVO group (*p* < 0.01, *p* < 0.001). These results prove that the MAPE system can effectively delay the oxidation rate of ATVO under light exposure, which might be attributed to the stable adsorption of modified amber at the oil-water interface [34]. 

These findings illustrate that MAPE offers superior protection against the oxidation of ATVO when exposed to light. Interestingly, the physical mixture group exhibited a lower MDA level compared to MAPE after 5 d, a reversal from the results at 1 and 3 d. This observation could potentially be explained by the self-emulsification of modified amber at the oil-water interface in a physical mixture under prolonged light exposure, leading to the relocation of MDA from the oil phase to the water phase [35].

#### 2.3.2. Analysis of Volatile Components

The total ion chromatogram of ATVO is depicted in Figure S1, showing clear peak separation and a smooth baseline. A total of 74 volatile components (Table S1) were detected in the ATVO, physical mixture, and MAPE groups after 1, 3, and 5 d of exposure to intense light. Differential components were identified by volcano plot using the Limma package, and 9, 18, and 20 differential components (Figure 2C) were filtered at d 1, 3, and 5, respectively. A total of 25 differential components were identified after consolidation and de-duplication. They were *α*-Phellandrene (000099-83-2), *α*-Terpinene (000099-86-5), Citronellal (000106-23-0), Myrcene (000123-35-3), *β*-Pinene (000127-91-3), *β*-Terpineol (000138-87-4), (E)-citral (000141-27-5), 2-Bornene (000464-17-5), (+)-delta-Cadinene (000483-76-1), Cyclene (000508-32-7), Terpinolene (000586-62-9), Sabinene (003387-41-5), Bicyclo[2.2.1]heptane (005794-04-7), (1R,5S)-1,8-Dimethyl-4-(propan-2-ylidene)spiro [4.5]dec-7-ene (028400-12-6), (+)-4-Carene (029050-33-7), *α*-Cuprenene (029621-78-1), (1S)-(-)-*α*-Pinene (007785-26-4), γ-Curcumene (997220-96-6), (+)-*α*-Pinene (007785-70-8), Geraniol (000106-24-1), *α*-Selinene (000473-13-2), 1-methyl-4-[(1R)-1,2,2-trimethylcyclope-ntyl] benzene (016982-00-6), (4E)-1-methyl-4-(6-methylhept-5-en-2-ylidene) cyclohexene (053585-13-0), *β*-selinene (017066-67-0), and (-)-*α*-muurolene (010208-80-7).

The cluster analysis of differential components in each group was visualized using the Pheatmap package. Samples were divided into two major groups (Figure 2D): (a) 1d-ATVO, 1d-physical mixture, and 1d and 3d-MAPE clustered together, suggesting less dissipation of volatile components in MAPE after 3 d of intense light exposure, with a similar content to that of ATVO treated for 1 d; (b) 3d and 5d-ATVO, 3d and 5d-physical mixture, and 5d-MAPE aggregated into another cluster, implying that MAPE still exerted a certain protective effect on volatile components after being treated for 5 d.

**Figure 3:**
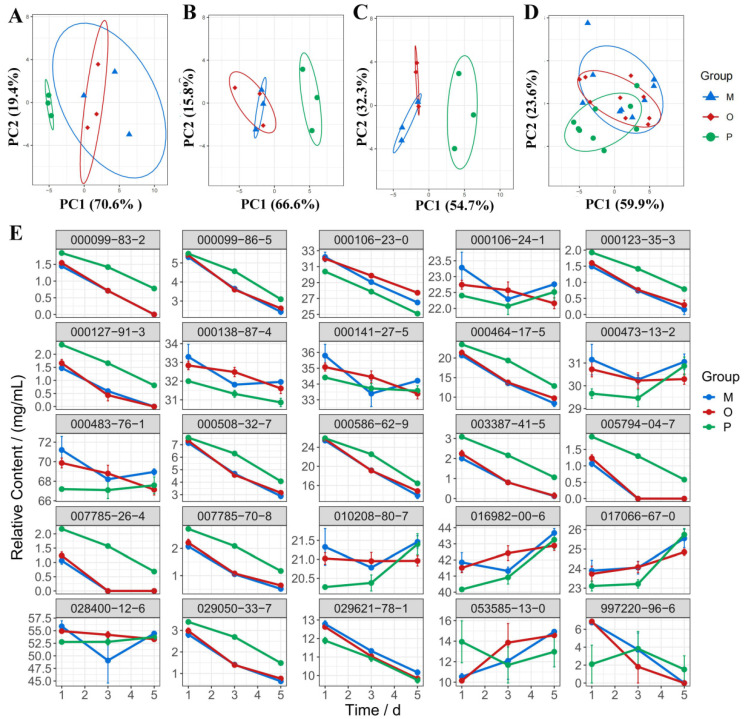
PCA and line chart of differential components. PCA plots of relative content of differential components in MAPE, physical mixture, and ATVO under intense light exposure at 1 d (**A**), 3 d (**B**), and 5 d (**C**), as well as the PCA plots of differential components in three groups at all time points (**D**). (**E**) Line chart depicting relative content of differential components in MAPE, physical mixture, and ATVO.

A PCA analysis of the differential components in each group was conducted to elucidate the differences between the groups. Results indicated that the sample distribution of the MAPE group did not overlap with that of the ATVO and physical mixture after treatment for 1, 3, and 5 d (Figure 3A–C), suggesting significant differences between MAPE and ATVO as well as physical mixtures. Furthermore, PC1 and PC2 contributed cumulatively to 90%, 82.4%, and 87%, respectively, proving that the principal components effectively retained the characteristics of the original data. Additionally, PCA analysis was performed on all samples. The sample distribution of the ATVO and physical mixture exhibited noticeable aggregation (Figure 3D), while there was only a partial overlap between MAPE and the ATVO and physical mixture, implying a unique expression of differential components in MAPE under intense light exposure.

Content change trends for differential components in each group, along with light exposure time, were created using the ggplot2 package. From Figure 3E, it was observed that the relative content of components such as 000099-86-5, 000106-24-1, 000123-35-3, 000141-27-5, 000464-17-5, 000483-76-1, 000508-32-7, 000586-62-9, 007785-70-8, 029050-33-7, and 029621-78-1 progressively decreased with increasing light exposure time in ATVO and physical mixture groups. Additionally, the relative content of components such as 000099-83-2, 000127-91-3, 003387-41-5, 005794-04-7, 007785-26-4, and 997220-96-6 even decreased to zero or near-zero over time in ATVO and physical mixture groups. However, MAPE played a role in mitigating the loss trend of these components under light exposure. Compared to the ATVO and physical mixture groups, the increase in the content of 053585-13-0 was delayed in MAPE. In summary, the MAPE system enhanced the stability of ATVO under light exposure conditions by slowing the loss trends of specific components and having less influence on other components. This enhancement was attributed to the closed-shell structure formed by the adsorbed modified amber particles at the oil-water interface.

#### 2.3.3. Analysis of the Volatilization Rules of Volatile Oil

**Figure 4:**
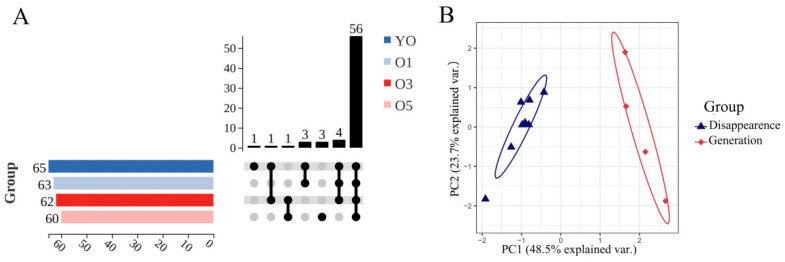
Physicochemical property visualization suite of qualitatively changed components. (**A**) UpSet plot of the volatile components in untreated ATVO and ATVO, which processed 1, 3, and 5 d of intense light exposure. (**B**) PCA plot of the physicochemical properties of the disappeared and generated components.

An UpSet analysis was conducted to identify qualitatively changed components. The untreated ATVO originally contained 65 volatile components, which gradually decreased with prolonged light exposure (Figure 4A). Specifically, two components disappeared after 1 d, four components vanished and one new component emerged after 3 d, and nine components disappeared while four new components appeared after 5 d. After organization and de-duplication, the nine vanished components were identified as 007785-26-4, 005794-04-7, 000127-91-3, 000099-83-2, 997220-96-6, 000500-00-5, 000110-93-0, 000124-18-5, and 007786-67-6, and the four newly generated components were 000536-60-7, 000502-47-6, 000481-34-5, and 043219-80-3. The content data are shown in Table S2. Some of the volatile components were more sensitive to light and disappeared from the free ATVO and the physical mixture after 1 d or 3 d of intense light exposure. This result suggests that light exposure may significantly affect the stability of volatile components and, thus, their bioactivity and pharmacological effects. The MAPE system was able to preserve six of those disappeared components in free ATVO and retard the generation rate of some components, which further demonstrates the potential of the MAPE system in enhancing the stability of ATVO.

Qualitatively changed components exhibited two distinctly different behavioral changes, raising questions about whether the intrinsic physicochemical properties of the volatile components contributed to these changes. An analysis was initiated by searching the physicochemical properties, including the relative molecular weight, melting point, boiling point, flash point, and LogP of the disappeared and generated components. However, the physicochemical property of component 997220-96-6 was unavailable and thus omitted from the analysis. PCA analysis based on the physicochemical parameters of the disappeared and generated components was conducted to explore the factors influencing their behavior. Figure 4B indicates a complete separation of the components that disappeared and those that were generated on PC1. Specifically, PC1 = 0.53 × relative molecular weight + 0.44 × density + 0.54 × boiling point + 0.41 × flash point − 0.24 × LogP, explaining 48.5% of the variance. PC1 showed a negative correlation with LogP and positive correlations with relative molecular weight, density, boiling point, and flash point, indicating that components with lower boiling points, flash points, relative molecular weights, and densities, as well as higher LogP, tended to evaporate under intense light conditions. The MAPE system helped mitigate the impact of the intrinsic physicochemical properties of volatile components to some extent, thereby enhancing the stability of ATVO.

### 2.4. In Vitro Release Studies of β-Asarone and α-Asarone from MAPE

**Figure 5:**
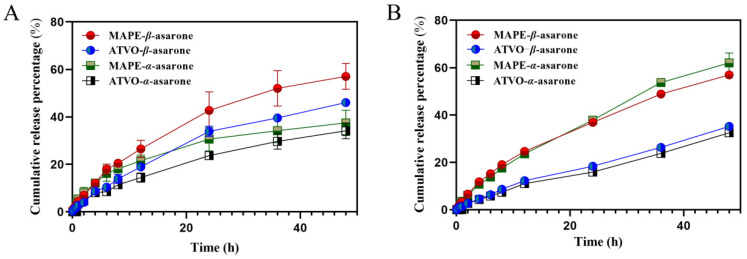
In vitro release of *β*-asarone and *α*-asarone from MAPE and ATVO in artificial gastric fluid (**A**) and artificial intestinal fluid (**B**).

The dialysis method has been widely employed in the field of drug release studies due to its straightforward experimental procedure and its ability to mimic physiological conditions [36]. The content determination method for *β*-asarone and *α*-asarone showed good performance in terms of specificity, standard curve, precision, reproducibility, stability, and recovery, as shown in Figure S2 and Tables S3–S7. Figure 5 illustrates the in vitro release curves of bioactive compounds from MAPE and ATVO in artificial gastric and artificial intestinal fluids. In artificial gastric fluid, the release rates of *β*-asarone and *α*-asarone in MAPE were faster within 24 h, followed by a slowing rise. By the end of 48 h, the difference in cumulative release rates of the two components in MAPE and ATVO was small. The cumulative release rate of *β*-asarone in MAPE was 57.07%, which was 1.24 times that of ATVO, while the cumulative release rate of *α*-asarone was 37.45%, or 1.10 times that of ATVO. In the artificial intestinal fluid environment, *β*-asarone and *α*-asarone in MAPE maintained a rapid release state. The cumulative release levels of 56.96% and 62.00% for *β*-asarone and *α*-asarone in MAPE after 48 h, respectively, were 1.62 and 1.91 times higher than those of ATVO. The higher cumulative release of *β*-asarone and *α*-asarone in MAPE suggests that Pickering emulsion technology could improve the in vitro release characteristics of ATVO.

### 2.5. In Vivo Bioavailability of the Loaded ATVO in MAPE

**Figure 6:**
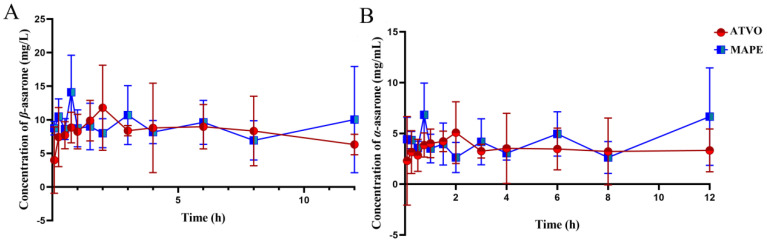
The blood *β*-asarone (**A**) and *α*-asarone (**B**) pharmaceutical kinetics of ATVO and MAPE in SD rats after oral administration.

Pickering emulsions are attracting attention in the biomedical and food fields for encapsulating lipophilic substances and enhancing the water solubility, stability, and bioavailability of lipophilic bioactive components [37]. To evaluate the effect of the Pickering emulsion system on improving the bioavailability and intrabiological behavior of ATVO, equal amounts of ATVO were encapsulated into MAPE and orally administered to SD rats. The blood concentrations of *β*-asarone and *α*-asarone were measured, and the methodological study of both compounds was presented in Figure S3 and Tables S8–S11. The plasma concentration-time curves of rats after gavage with MAPE and ATVO are shown in Figure 6. The pharmacokinetic parameters were fitted by DAS 2.0 software, and the results are presented in Table 1. Figure 6 shows that the blood concentrations of *β*-asarone and *α*-asarone in the MAPE group were significantly higher than those in the ATVO group during the first hour, and the concentrations remained higher at 3 h and 6 h. Furthermore, it was observed that after 9 h, the blood concentrations of *β*-asarone and *α*-asarone in the MAPE group exhibited an increasing trend. These findings signify that Pickering emulsion technology could accelerate the entry of *β*-asarone and α-asarone into the blood to some extent.

The pharmacokinetic parameters in Table 1 indicate that the T_1/2_ and T_max_ of *β*-asarone in MAPE were 0.65 and 1.41 times that of ATVO, respectively, while the T_1/2_ and T_max_ of *α*-asarone were 1.27 and 0.70 times that of ATVO. Additionally, the elimination rate of *α*-asarone in MAPE was slower, while the elimination rate of *β*-asarone was faster than that in ATVO. The above parameters indicate that there are differences in the in vivo absorption and elimination rates of free ATVO and the loaded ATVO in MAPE. The C_max_, AUC_0–t_, and AUC_0–∞_ of *β*-asarone in MAPE were 1.12, 1.03, and 1.11 times that of ATVO, separately; for *α*-asarone, they were 1.27, 1.22, and 1.11 times that of ATVO. These results indicate that the oral bioavailability of *β*-asarone and α-asarone was enhanced after existing in Pickering emulsion formulation, especially for *α*-asarone, possibly because MAPE has better release in the intestinal environment. These results implied a certain correlation between drug release patterns and formulation form. Liu et al. [38] conducted a pharmacokinetic study on enteric-coated capsules loaded with curcumin Pickering emulsion powder and found that the bioavailability of curcumin in four α-lactalbumin-stabilized PE was higher than that of unencapsulated curcumin, with the highest bioavailability of NSs-PE being 5.37 times that of unencapsulated curcumin. In conclusion, the efficacy of MAPE in improving the bioavailability of *β*-asarone and α-asarone was limited, possibly due to the micrometer-sized emulsion droplets and the influence of the gastrointestinal environment in rats on the structure of MAPE.

## 3. Discussion

In this study, we stabilized the *Acorus tatarinowii* volatile oil Pickering emulsion using modified Chinese medicinal solid powder and modified amber particles and further investigated the stability, in vitro release behavior, and pharmacokinetics of the emulsion. The use of the medicinal powder inherently contained in Chinese medicinal preparations to solve the limitations of the preparation itself aligns with the concept of ‘integrating drug and excipient’ in traditional Chinese medicine. This study provides an innovative approach with characteristics of traditional Chinese medicine to improving the stability of volatile oils.

We have noticed that the aggregated particles (modified amber) shown in Figure 1C were characterized by a size of tens of micrometers, similar to the droplets in Figure 1E. The aggregated modified amber particles might have been broken into a size suitable for stabilizing the emulsion under the strong mechanical force of high-speed shear. The individual free particles, stained red by Nile blue and with a particle size much smaller than 10 μm, can be clearly observed in the confocal laser microscopy images (Figure 1E), which further supports this assumption.

Additionally, the microscopic images of the MAPE system show that the system mainly exhibited an O/W type but also possessed the characteristics of W/O/W. This may be due to several reasons: some of the local area of modified amber particles retained the lipophilicity of amber itself, and this property made it potentially stabilize both oil-in-water and water-in-oil interfaces simultaneously. In W/O/W systems, this lipophilicity facilitates the aggregation of some modified amber particles at the inner interface, resulting in an obvious fluorescence. Secondly, in the MAPE system, the percentage of the oil phase is as high as 65%. Relevant studies [39] have shown that an increase in oil content significantly increases the number of W/O droplets in W/O/W double emulsions and the size of oil droplets. In the MAPE system, a higher oil content implies that W/O droplets may form when amphiphilic modified amber particles are present, and the aqueous phase will encapsulate these droplets during the subsequent shear process, thereby forming W/O/W emulsions. In addition, a study on W_1_/O/W_2_ emulgels stabilized by *Sipunculus nudus* salt-soluble proteins [40] reported that the degradation of vitamin C encapsulated in the W_1_ phase and *β*-carotene in the oil phase has been effectively prevented, thereby maintaining a high level of antioxidant capacity. Therefore, it is hypothesized that this distribution of modified amber particles would not affect the stability of the emulsion or the stability of the active ingredient.

During the stability analysis, our results revealed that the MAPE system was able to inhibit the oxidation rate of the ATVO in a light environment and maintain both the composition and content of the volatile components in the ATVO. Meanwhile, a further analysis of the physicochemical properties of the volatile components that underwent qualitative change behaviors implied that those compounds with lower boiling points, flash points, relative molecular weights, densities, and higher LogP tended to volatilize under the light environment. This conclusion is consistent with the view presented in a related reference that volatile compounds are low-molecular-weight molecules that readily evaporate at room temperature due to their low boiling points [41].

The water insolubility of volatile oils limits their further application and clinical efficacy to a certain extent [42]. Horváth et al. [43] utilized modified silica nanoparticles as stabilizers to prepare tea tree and thyme volatile oils and investigated their in vitro release properties. Results revealed that PE exhibited greater oil loading capacity and improved in vitro diffusion properties compared to traditional emulsions stabilized with Tween 80. In addition, further research by Horváth et al. [44] revealed that the Pickering emulsion system of volatile oils stabilized by modified silica nanoparticles exhibited significantly better antibacterial activity compared to traditional emulsions stabilized with Tween 80 and volatile oil ethanol solutions, particularly cinnamon volatile oil PE. This finding suggests that the pharmacological effects of volatile oils are closely linked to their release characteristics. Specifically, the enhanced release properties may contribute to the increased bioactivity of volatile oils, thereby improving their effectiveness in antibacterial and other medical applications.

The emulsification properties of solid particles are easily affected by external environmental factors such as pH, thus affecting the stability of Pickering emulsion. Yang et al. [45] found that, in the absence of NaCl, a stable gel-like high internal phase Pickering emulsion could only be formed within the pH range of 5.0 to 10.0. Chitosan-stabilized O/W Pickering emulsions exhibit high stability at pH 7 and above [46]. However, the PE system demonstrated instability, including droplet coalescence and creaming, as the environmental pH gradually decreased. In particular, the demulsification phenomenon was observed at a pH of 2. In addition, some starch-based Pickering emulsions exhibited reduced stability in a more acidic environment (pH < 4), due to an excess of hydrogen ions that could impede the swelling of starch particles, which prevents the formation of a stable gel structure [47]. After 24 h and beyond, the standard deviations of *β*-asarone and *α*-asarone in the MAPE system under artificial gastric fluid conditions significantly increased. This increase may be attributed to the prolonged exposure to an acidic environment, which significantly affects the emulsifying properties of the modified amber particles. Such an impact could lead to increased aggregation and interactions among the particles, thereby weakening the uniformity and stability of the emulsion. Consequently, the release behavior of β-asarone and α-asarone in this environment may also become unstable.

The standard deviations of some pharmacokinetic parameters for *β*-asarone and *α*-asarone in the MAPE and ATVO groups in Table 1 were relatively high. In this study, the 12 rats in each group were divided into two parts, and blood samples were alternately collected at each time point. This alternating blood sampling design may be influenced by the inherent differences between the two groups of rats, thereby reducing the coherence of the pharmacokinetic data, as evidenced by the fluctuations in the pharmacokinetic curves. This may affect the accuracy of the pharmacokinetic parameter data. Despite these fluctuations, the specific data indicate that the blood concentrations of *β*-asarone and *α*-asarone in the MAPE group were higher than those in the ATVO group for most of the time points. This result is consistent with the in vitro release study data, suggesting that the PE system can effectively enhance the diffusion rate of the volitale oils, facilitating their entry into the bloodstream and thereby increasing their bioavailability.

## 4. Materials and Methods

### 4.1. Materials

Amber was provided by Lei Yun Shang Pharmaceutical Group Co., Ltd. (Suzhou, China). ATVO was purchased from Xian Deshengyuan Biotechnology Co., Ltd. (Xi’an, China). Nile Red was provided by CSNpharm (Chicago, IL, USA), and Nile Blue was purchased from Sigma-Aldrich (Shanghai) Trading Co., Ltd. (Shanghai, China). *β*-asarone and *α*-asarone standards were acquired from Shanghai Yuanye Biotechnology Co., Ltd. (Shanghai, China). Pepsin, trypsin, and n-Docosane (98%) were purchased from Shanghai Macklin Biochemical Technology Co., Ltd. (Shanghai, China). All other reagents were of analytical grade.

### 4.2. Modification of Amber with PEG6000

Amber is a W/O type stabilizer, and it is intended to be designed as an O/W type stabilizer through surface modification techniques. Initially, 2 g of PEG6000 were placed in a 100 mL evaporation vessel on an electric heating mantle at a voltage of 150 volts, which preheated for 10 min, until completely dissolved. Subsequently, 4 g of amber powder were swiftly added and stirred vigorously with a glass rod for 5 min to ensure thorough mixing. The modified amber was then obtained after being left to dry at room temperature for 24 h.

### 4.3. Encapsulation of ATVO in Pickering Emulsion

ATVO in Lingzhu Pulvis served as the oil phase. Initially, 65% *v*/*v* of ATVO was added into a 50 mL test tube as the dispersed phase, followed by the addition of the aqueous phase and modified amber particles at a concentration of 7.5 mg/mL. The two phases were subsequently homogenized using the IKA T18 high shear dispersion machine (IKA, Staufen im Breisgau, Germany) at 11,000 rpm for 2 min to generate the Pickering emulsion. The entire emulsification process was conducted at room temperature, and the resulting emulsion was designated as MAPE. The prepared emulsions were stored at room temperature for subsequent analysis.

### 4.4. Characterization of Modified Amber and MAPE

The Fourier Transform Infrared (FTIR) spectroscopy of modified amber particles was analyzed using a Bruker Tensor 27 FTIR spectrometer (Bruker, Berlin, Germany), with a resolution of 4 cm^−1^ and a wavelength range from 4000 to 400 cm^−1^. The particle morphology was observed using GeminiSEM360 (ZEISS, Jena, Germany). Initially, a small amount of modified amber particles and PEG6000 powder were each adhered to conductive gel, gold-coated under vacuum conditions, and then fixed to sample holders for imaging. The wettability of modified amber and amber was carried out with an optical contact angle device (LSA100, LAUDA Scientific, Lauda-Königshofen, Germany). The emulsion type of PE was determined through droplet dilution experiments. The droplet size of MAPE was measured using a Microtrac S3500 laser particle size analyzer (Microtrac Inc., St A, Largo, FL, USA). A Confocal Laser Scanning Microscope (CLSM) was employed to observe the MAPE sample, allowing for high-resolution imaging of its structural characteristics and the distribution of oil phases. The MAPE was mixed with mixed fluorescence dyes (Nile Red isopropanol solution and Nile Blue aqueous solution). The images of the stained emulsion were captured by a SP8 DIVE multiphoton laser scanning confocal microscope (Leica Microsystems, Wetzlar, Germany). Nile Red was used for staining the oil phase (marked in green), excited at 488 nm; Nile Blue was used for the stained modified amber-colored particles (marked in red), excited at 633 nm.

### 4.5. Stability Study of ATVO in MAPE under Strong Lighting

To assess the stability of the loaded ATVO in the MAPE system, we studied the stability of the ATVO under light exposure conditions. The stability analysis was carried out by placing the MAPE, free ATVO, and oil-water mixture in a light environment (the light intensity was 4500 ± 500 lx). Three samples from each group were collected on 1, 3, and 5 d, respectively. The samples from MAPE and physical mixture groups were centrifuged at 10,000 rpm for 20 min to separate the upper oil layer. The MDA content in each sample was determined using the spectrophotometric method specified in GB5009.181-2016 [48] with slight modifications, and the PV was determined using the titration method specified in GB 5009.227-2016 [49]. Simultaneously, the volatile components of ATVO in each group were analyzed using the Agilent 7890B/5977B GC-MS analyzer (Agilent Technologies, Santa Clara, CA, USA) based on previous studies [23], aiming to examine the effect of the PE system on improving the stability of ATVO from the perspective of changes in volatile component content and composition.

After GC-MS data acquisition, Data Analysis software was used to identify the volatile components in each group by calling the W11N17main.L database. RStudio software (2022.02.0), with the help of the dplyr package (1.0.10) [50], was used to select the peak area and CAS number of volatile components with a matching degree ≥ 60, then calculated and summarized the relative content of volatile components by the internal standard method. The Limma package (3.50.3) [51] was utilized to compare the relative content of volatile components in free ATVO samples without light exposure and those free ATVO exposed to light for 1, 3, and 5 d, and a threshold of logFC > 1.5 and *p* < 0.05 was used to select differential components. The Pheatmap package (1.0.12) [52] was used to visualize the expression of differential component content in each group. Principal component analysis (PCA) of the differential components in each group was performed using the ggbiplot package (0.55) [53]. The line chart showing the changes in the relative content of differential components during the light exposure was created using the ggplot2 [54] package to visualize the trends in component content. The UpSet analysis function on the OmicShare Tools platform (https://www.omicshare.com/tools) was used to screen qualitatively changed components between the three groups exposed to light for 5 d and the ATVO that did not receive light treatment (accessed on 18 May 2024).

### 4.6. In Vitro Release Studies of MAPE

The release behavior of loaded ATVO from MAPE and free ATVO was evaluated through the dialysis method by simulating the physiological pH and composition of gastric and intestinal fluids [55,56]. Firstly, ATVO and laboratory-made MAPE were transferred to wet dialysis bags with a molecular weight cut-off of 300 kDa. The dialysis bags were then suspended in 200 mL of artificial gastric fluid or artificial intestinal fluid and placed in a shaking water bath at 37 °C with a speed of 100 rpm. These operations were conducted in parallel three times for each group. Timing started when the dialysis bags came into contact with the release medium. At specified time points of 0.08, 0.25, 0.5, 0.75, 1, 2, 4, 6, 8, 12, 24, 36, and 48 h, 2 mL of the released solution was taken, and an equal volume of fresh release medium was added. The collected solutions were mixed with 2 mL of n-hexane, respectively, vortexed for 3 min, and allowed to stand. Then, the upper n-hexane layer was collected and dehydrated with anhydrous sodium sulfate. Finally, the filtrate was passed through a 0.22 μm microporous membrane and analyzed by GC-MS to determine the content of *β*-asarone and *α*-asarone in the released solution. The cumulative release percentages of *β*-asarone and *α*-asarone at each time point were calculated according to Formula (1).

(1) $$Cumulative\;release\;percentage\;(\%)=\frac{\left[C_{n}V_{1}+\left(C_{n-1}+C_{n-2}+\cdots +C_{2}+C_{1}\right)V_{2}\right]}{W}\times 100\%$$

C_n_ is the concentration of *β*-asarone and *α*-asarone in the release medium at the nth sampling point (µg/mL); V_1_ is the total volume of the release medium (mL); V_2_ is the sampling volume (mL); and W is the theoretical maximum solubility of *β*-asarone and *α*-asarone in the release medium (µg). The determination method and detailed information on the maximum solubility of *β*-asarone and *α*-asarone could be found in supplement materials.

### 4.7. Pharmacokinetics of Pickering Emulsion-Loaded ATVO

Thirty-six SD male rats weighing 180–220 g were purchased from Chengdu Dashuo Experimental Animal Co., Ltd. (Chengdu, China) and had license number SCXK (Sichuan) 2020–030. Thirty-six rats were randomly divided into three groups and housed in the Laboratory of Pharmacology of Chinese Medicine at Shaanxi University of Chinese Medicine for 7 d. After fasting for 12 h (water was allowed), the ATVO group and MAPE group were administered via gavage to SD rats at a dose of 752 mg/kg of ATVO. Blood samples (0.5 mL) were alternately taken from six rats by the orbital vein at 0.08, 0.25, 0.5, 0.75, 1, 1.5, 2, 3, 4, 6, 8, and 12 h after administration. The experiment was approved by the Animal Ethics Committee of Shaanxi University of Chinese Medicine, with the approval number SUCMDL20240226005. The blood samples were centrifuged at 4000 rpm for 10 min, and the supernatant serum was obtained and stored at −80 °C. 150 μL of plasma sample was collected, and 150 μL of n-hexane was added, subsequently vortexed for 5 min. The mixture was centrifuged at 4 °C and 12,000 rpm for 10 min to obtain the supernatant for analysis. The concentration of *β*-asarone and *α*-asarone was measured by GC-MS with high reliability.

### 4.8. Statistical Analysis

The data were denoted as mean ± sd ($\bar{x}$ ± sd). Statistical significance among the MAPE, ATVO, and physical mixture groups was assessed using One-way analysis of variance (ANOVA), followed by a Tukey post-test with SPSS 26 software. The level of statistical significance was determined as follows: * *p* < 0.05, ** *p* < 0.01, and *** *p* < 0.001.

## 5. Conclusions

Modified amber particles with suitable wettability were successfully prepared through the melt method using PEG6000, and the MAPE system exhibited good antioxidant stability of ATVO under strong light. Further analysis of the volatility pattern revealed that volatile components with lower boiling points, flash points, relative molecular weights, and densities, as well as higher LogP, tended to volatilize under strong light conditions. The release behavior of *β*-asarone and *α*-asarone from MAPE was characterized by rapid and sustained release in the simulated gastric and intestinal environments. And the in vivo pharmacokinetic behavior of the MAPE system loaded with ATVO differs slightly from that of free ATVO, showing better bioavailability. This study demonstrated that the PE system containing volatile oils could be an effective drug system for improving the stability of volatile oils and provided a reference for the enhancement of volatile oil stability in oil-containing Chinese medicine solid formulations.

## Figures and Tables

**Table 1:** Pharmacokinetic parameters of *β*-asarone and *α*-asarone after oral administration of ATVO and MAPE formulation (n = 6, $\bar{x}$ ± sd).

Pharmacokinetic Parameters	*β*-Asarone		*α*-Asarone	
	ATVO	MAPE	ATVO	MAPE
*T*_1/2_ (h)	14.30 ± 17.09	9.33 ± 5.67	7.76 ± 4.18	9.85 ± 5.68
*T*_max_ (h)	2.46 ± 2.79	3.46 ± 4.70	3.35 ± 3.67	2.35 ± 4.74
*C*_max_ (mg·L^−1^)	14.94 ± 4.55	16.67 ± 6.04	6.82 ± 2.67	8.63 ± 3.75
MRT_0–t_ (h)	5.59 ± 0.34	5.82 ± 0.67	5.25 ± 1.86	6.45 ± 0.85
MRT_0–∞_ (h)	21.77 ± 22.64	15.56 ± 7.67	12.33 ± 5.76	18.41 ± 5.69
AUC_0–t_ (mg·L^−1^·h)	102.77 ± 29.79	105.81 ± 26.10	44.87 ± 21.56	54.94 ± 25.38
AUC_0–∞_ (mg·L^−1^·h)	228.70 ± 118.32	253.20 ± 150.90	111.71 ± 51.30	123.50 ± 42.91

## Data Availability

The study data are contained within the article and Supplementary Materials.

## Supplementary Materials

The following supporting information can be downloaded at: https://www.mdpi.com/article/10.3390/ph17091117/s1: Figure S1: GC-MS total ion flow diagram of ATVO; Figure S2: GC-MS chromatogram of *β*-asarone and *α*-asarone; Figure S3: Chromatogram of plasma sample; Table S1: Relative content of 74 volatile components detected in ATVO, physical mixture, and modified amber-PE after 1, 3, and 5 d of exposure to intense light ($\bar{x}$ ± sd, n = 3); Table S2: Relative content of qualitatively changed components detected in ATVO, physical mixture, and MAPE after 1, 3, and 5 d of exposure to intense light ($\bar{x}$ ± sd, n = 3); Table S3: Standard curves of *β*-asarone and *α*-asarone; Table S4: Precision of *β*-asarone and *α*-asarone; Table S5: Reproducibility of *β*-asarone and *α*-asarone; Table S6: Stability of *β*-asarone and *α*-asarone; Table S7: Recoveries of *β*-asarone and *α*-asarone; Table S8: Standard curves of *β*-asarone and *α*-asarone in plasma; Table S9: Intra-day and inter-day precision of *β*-asarone and *α*-asarone in rat plasma; Table S10: Extraction recovery and matrix effects of *β*-asarone and *α*-asarone in rat plasma; Table S11: Stability of *β*-asarone and *α*-asarone in rat plasma; Table S12: Blood-drug concentration of *β*-asarone and *α*-asarone in MAPE and ATVO.
