# Supplementary material for: Development, Stability, and In Vitro/In Vivo Studies of Volatile Oil Pickering Emulsion Stabilized by Modified Amber
Source: Pharmaceuticals (Basel). 2024 Aug 24;17(9):1117. doi: 10.3390/ph17091117 (PMC11434788; doi:10.3390/ph17091117)
Supplement: Supplementary file 1 [file pharmaceuticals-17-01117-s001.zip › pharmaceuticals-3121960-supplementary.pdf]

Supplementary Materials

Development, Stability, and In Vitro/In Vivo Studies of Volatile Oil Pickering Emulsion Stabilized by Modified Amber

Maomao Zhu [1,†], Zhonghuan Qu [2,†], Yanjun Yang [1], Ruyu Shi [1], Bing Yang [1], Yajun Shi [2], Junbo Zou [2,*] and Xiaobin Jia [1,*]

[1] Innovation Center for Industry-Education Integration of Pediatrics and Traditional Chinese Medicine, State Key Laboratory of Natural Medicines, School of Traditional Chinese Pharmacy, China Pharmaceutical University, Nanjing 211198, China; 13675131107@163.com (M.Z.); 15706037600@163.com (Y.Y.); s951001@126.com (R.S.); 15751151582@163.com (B.Y.)

[2] Shaanxi Province Key Laboratory of New Drugs and Chinese Medicine Foundation Research, College of Pharmacy, Shaanxi University of Chinese Medicine, Xianyang 712046, China; zhq19980221@163.com (Z.Q.); 2051004@sntcm.edu.cn (Y.S.)

* Correspondence: 2051078@sntcm.edu.cn (J.Z.); jiaxiaobin2015@163.com (X.J.); Tel.: +86-153-1907-0696 (J.Z.); +86-136-0515-7558 (X.J.)

† These authors contributed equally to this work.

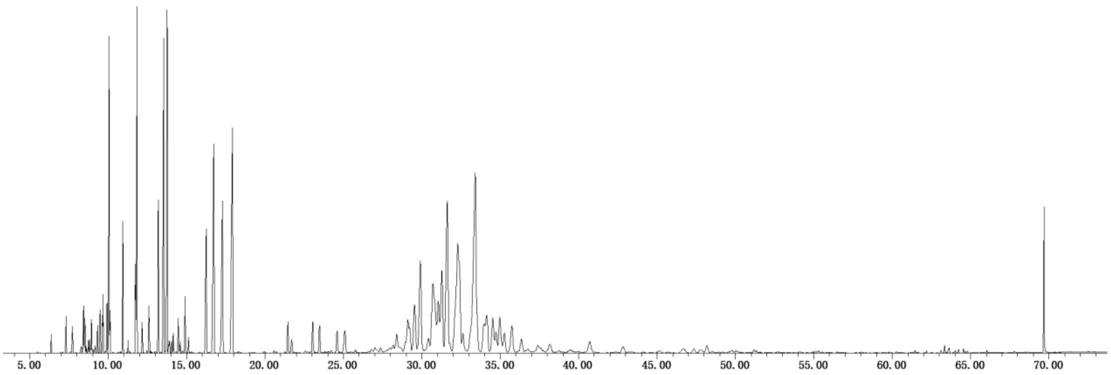

Figure S1 GC-MS total ion flow diagram of ATVO

Table S1 Relative content of 74 kind volatile components detected in ATVO, physical mixture, and MAPE after 1, 3, and 5 d of exposure to intense light ($\bar{x}$±sd, n=3)

CAS	Compound name	Untreated ATVO	1d-M	1d-O	1d-P	3d-M	3d-O	3d-P	5d-M	5d-O	5d-P
007785-26-4	(1S)-(-)-alpha-Pinene	2.1473±0.1305	1.0637±0.2156	1.2388±0.2242	2.1743±0.0242	0.0000±0.0000	0.0000±0.0000	1.5709±0.1182	0.0000±0.0000	0.0000±0.0000	0.6827±0.1105
005794-04-7	Bicyclo[2.2.1]heptane	1.8177±0.1045	1.0677±0.1546	1.2313±0.1847	1.8902±0.0146	0.0000±0.0000	0.0000±0.0000	1.2976±0.0849	0.0000±0.0000	0.0000±0.0000	0.5807±0.1049
003387-41-5	Sabinene	3.0682±0.1753	2.0106±0.2277	2.2447±0.2955	3.0878±0.0336	0.8133±0.1000	0.8084±0.1741	2.1622±0.1635	0.1278±0.2213	0.1442±0.2497	1.0650±0.1080
000127-91-3	Beta-Pinene	2.3273±0.1394	1.4715±0.1730	1.6591±0.2524	2.3675±0.0214	0.5904±0.0758	0.4356±0.3776	1.6582±0.1415	0.0000±0.0000	0.0000±0.0000	0.8106±0.0813
000500-00-5	p-Menth-3-Ene	0.8131±0.0442	0.3924±0.3423	0.6200±0.0708	0.8369±0.0106	0.0000±0.0000	0.0000±0.0000	0.6405±0.0408	0.0000±0.0000	0.0000±0.0000	0.0000±0.0000
000110-93-0	6-Methyl-5-hepten-2-one	0.7945±0.0499	0.7313±0.0304	0.7610±0.0434	0.7461±0.0012	0.4886±0.0291	0.1785±0.3091	0.1842±0.3190	0.0000±0.0000	0.0000±0.0000	0.0000±0.0000
000123-35-3	Myrcene	1.9869±0.1171	1.4905±0.1152	1.5979±0.1345	1.9273±0.0107	0.7385±0.0736	0.7637±0.0915	1.4180±0.0736	0.1530±0.2650	0.2944±0.2595	0.7879±0.0383
000124-18-5	Decane	0.3606±0.3129	0.0000±0.0000	0.0000±0.0000	0.0000±0.0000	0.0000±0.0000	0.0000±0.0000	0.0000±0.0000	0.0000±0.0000	0.0000±0.0000	0.0000±0.0000
000099-83-2	α-Phellandrene	1.9377±0.1024	1.4460±0.1204	1.5423±0.1315	1.8412±0.0373	0.7100±0.0680	0.7113±0.0835	1.4183±0.0746	0.0000±0.0000	0.0000±0.0000	0.7774±0.0567
007785-70-8	(1R)-(+)-alpha-Pinene	2.6663±0.1389	2.0713±0.1775	2.2091±0.2154	2.7154±0.0226	1.0533±0.1035	1.0807±0.1286	2.0863±0.1045	0.5149±0.1345	0.6403±0.0776	1.1718±0.0790
000470-67-7	1,4-Cineole	2.2515±0.1170	2.0506±0.0971	2.1242±0.1482	2.0855±0.0160	1.3251±0.0826	0.8744±0.7610	1.3551±0.2116	0.5996±0.5344	0.9833±0.0823	0.7140±0.1122
029050-33-7	(+)-4-Carene	3.8175±0.2250	2.8078±0.2387	2.9766±0.2308	3.3923±0.0893	1.4107±0.1132	1.3942±0.1356	2.6947±0.1502	0.6369±0.1404	0.7636±0.0854	1.4846±0.1345
000099-87-6	p-Cymene	3.4153±0.2036	4.1616±0.0610	4.2938±0.2598	4.9071±0.1338	3.3083±0.2176	3.6287±0.2670	3.5886±0.1775	2.7028±0.3970	3.1534±0.2027	2.8753±0.1362
000464-17-5	2-Bornene	22.0387±1.5453	20.6425±0.9062	21.3400±1.2239	23.4698±0.0382	13.5439±0.8166	13.7534±1.0385	19.3612±0.7015	8.4247±1.4179	9.7297±0.8120	12.8546±0.4837
000470-82-6	Cineole	2.2295±0.1302	2.1189±0.0768	2.1809±0.1197	2.1332±0.0158	1.4827±0.0808	1.5180±0.0990	1.5127±0.1832	1.0833±0.1363	1.1743±0.0752	0.9047±0.1057
000508-32-7	Cyclene	8.3289±0.4572	7.1430±0.3579	7.3218±0.3627	7.5699±0.1703	4.6804±0.2154	4.5791±0.2567	6.2978±0.2375	2.8822±0.3514	3.1472±0.2263	4.0708±0.1799
000586-67-4	1-Isopropenyl-4-Methylcyclohexene	0.8879±0.0517	0.6932±0.1193	0.8356±0.0376	0.8874±0.0091	0.4335±0.0110	0.4428±0.0196	0.6935±0.0257	0.1080±0.1871	0.1180±0.2045	0.3805±0.0076
000099-86-5	alpha-Terpinene	5.9009±0.3518	5.3052±0.2277	5.3993±0.2213	5.4839±0.1332	3.6462±0.1391	3.5980±0.1444	4.5636±0.1582	2.4347±0.2426	2.6129±0.1625	3.0943±0.1007
000586-62-9	Terpinolene	26.9609±1.4870	25.4007±0.8124	25.7293±0.9371	25.8813±0.2871	19.1659±0.6541	19.1124±0.6439	22.5416±0.6484	13.8989±1.1553	14.8129±0.7748	16.4339±0.3880
000078-70-6	Linalool	2.0281±0.1267	2.2300±0.0820	2.2043±0.0348	2.1467±0.0069	2.0373±0.0101	2.0930±0.0231	1.9939±0.0195	2.0220±0.0364	2.0351±0.0368	1.8878±0.0296
001632-73-1	(1R)-(+)-Fenchyl alcohol	3.1732±0.1845	3.5240±0.1234	3.4850±0.0557	3.4072±0.0135	3.2392±0.0288	3.3178±0.0422	3.1616±0.0363	3.2139±0.0352	3.1856±0.0633	3.0224±0.0370

000586-82-3	3-Cyclohexen-1-ol,1-methyl-4-(1-methylethyl)-	11.1886±0.6448	12.4806±0.4274	12.3437±0.2075	11.9753±0.0223	11.6486±0.0966	11.8348±0.1229	11.3966±0.0980	11.5771±0.0727	11.4667±0.1900	11.0937±0.1096
000138-87-4	1-Methyl-4-Prop-1-En-2-Ylcyclohexan-1-Ol	30.1133±1.6101	33.2943±1.1579	32.8370±0.3953	31.9991±0.1035	31.8101±0.1757	32.4846±0.4324	31.3197±0.3263	31.9565±0.1611	31.6117±0.4759	30.8628±0.3812
000106-23-0	Citronellal	31.2055±1.7010	32.2112±1.0308	31.9943±0.5035	30.3817±0.2039	29.0606±0.2902	29.8660±0.3685	27.8581±0.2480	26.4953±0.3920	27.7201±0.5050	25.0939±0.1779
000124-76-5	DL-Isoborneol	1.2697±0.3949	1.7772±0.0912	1.7797±0.0264	1.3386±0.3647	1.4493±0.3401	1.4728±0.3271	1.4640±0.3486	1.2902±0.3271	1.5964±0.0247	1.4498±0.3069
000464-45-9	L(-)-Borneol	1.4348±0.0764	1.6021±0.0646	1.5884±0.0200	1.5312±0.0179	1.4951±0.0223	1.5235±0.0288	1.4876±0.0186	1.5288±0.0064	1.4924±0.0179	1.4818±0.0231
000562-74-3	Terpinen-4-ol	2.3493±0.1420	2.6150±0.0948	2.5471±0.0367	2.5370±0.0168	2.4204±0.0290	2.4752±0.0332	2.4365±0.0271	2.4303±0.0373	2.3885±0.0527	2.3424±0.0403
055722-59-3	Isocitral	0.8113±0.0542	0.7944±0.0158	0.7714±0.0259	0.7553±0.0041	0.6722±0.0263	0.6430±0.0144	0.6803±0.0116	0.5972±0.0318	0.3791±0.3284	0.6065±0.0155
000098-55-5	alpha-Terpineol	4.1159±0.2332	4.6493±0.1685	4.5733±0.0618	4.4566±0.0099	4.3890±0.0146	4.4782±0.0640	4.3011±0.0572	4.4697±0.0149	4.4055±0.0615	4.3290±0.0716
000586-81-2	gamma-Terpineol	1.1911±0.0855	1.3116±0.0302	1.2848±0.0197	1.2499±0.0073	1.2025±0.0105	1.2356±0.0189	1.1775±0.0141	1.2059±0.0068	1.1968±0.0184	1.1719±0.0156
001117-61-9	(R)-Citronellol	13.8137±0.7732	15.6450±0.5643	15.2363±0.1602	15.0413±0.0237	14.9083±0.0501	15.0675±0.3145	14.7133±0.2990	15.2615±0.1180	14.7882±0.1936	15.0144±0.3481
000106-26-3	cis-Citral	27.4945±1.7753	28.3005±0.9159	27.7961±0.2713	27.0973±0.1768	26.6498±0.1065	26.9343±0.4928	26.3378±0.4215	26.3530±0.0743	25.9966±0.4873	25.5982±0.3110
000106-24-1	Geraniol	20.6478±1.1328	23.2841±0.8381	22.7453±0.2427	22.4033±0.0348	22.2923±0.0843	22.5714±0.4511	22.0726±0.4563	22.7609±0.1006	22.1597±0.2941	22.5159±0.5124
000141-27-5	trans-3,7-Dimethyl-Octa-2,6-Dien-1-Al	32.3704±1.7370	35.7999±1.2378	35.0767±0.4429	34.4155±0.0998	33.3942±1.4193	34.4532±0.6518	33.7223±0.5425	34.2113±0.1007	33.3909±0.5753	33.5757±0.5451
002792-39-4	(6E)-2,6-Dimethylocta-2,6-Diene	3.2068±0.1862	3.7523±0.1522	3.5993±0.0413	3.5749±0.0062	3.5551±0.0306	3.5516±0.0760	3.4870±0.0508	3.5749±0.0034	3.5322±0.0403	3.5558±0.0748
000097-53-0	Eugenol	1.6054±0.0846	1.8602±0.1062	1.7867±0.0197	1.6307±0.0222	1.6734±0.0639	1.7313±0.1191	1.4047±0.0513	1.0553±0.0230	1.2749±0.3505	0.8163±0.0386
000141-12-8	Neryl acetate	3.8068±0.2321	4.4050±0.2216	4.3382±0.1369	4.2578±0.0077	4.2033±0.0302	4.1948±0.0297	4.1214±0.0497	4.4024±0.0598	4.1971±0.1048	4.3256±0.0429
000515-13-9	beta-Elemen	3.5601±0.2158	4.0138±0.1563	3.9235±0.0497	3.8064±0.0012	3.7922±0.0146	3.8044±0.0915	3.7098±0.0560	3.8461±0.0066	3.7280±0.0860	3.7640±0.0732
000469-61-4	alpha-Cedrene	3.1895±0.1816	3.6948±0.1350	3.6299±0.0454	3.5046±0.0188	3.4792±0.0122	3.5375±0.0624	3.4110±0.0413	3.5474±0.0195	3.5123±0.0546	3.4632±0.0692
000546-28-1	(+)-beta-Cedrene	4.5133±0.2537	5.1990±0.1396	5.0753±0.0724	4.9265±0.0173	4.9202±0.0423	4.9588±0.0736	4.8423±0.0775	4.9721±0.0587	4.8752±0.0741	4.8527±0.0803
094535-52-1	(1R,3aS,4aS,8aS)-1,4,4,6-tetramethyl-1,2,3,3a,4,4a,7,8-octahydrocyclopenta[1,4] cyclobuta[1,2]benzene	4.0523±0.9534	3.4099±0.1368	4.0072±1.2746	4.2178±1.8247	4.8267±1.6976	4.8949±1.2987	3.1945±0.0240	4.8763±1.4227	4.1123±1.0783	5.9169±0.2634
103827-22-1	4a,8-Dimethyl-2-(prop-1-en-2-yl)-1,2,3,4,4a,5,6,7-octahydronaphthalene	8.0914±0.1705	8.3628±0.4104	8.8362±0.6721	8.3154±0.9066	8.7608±0.6888	8.6438±0.6494	8.0128±0.0273	8.9390±0.7135	8.2693±0.4317	9.2626±0.3394
030021-74-0	gamma-muurolene	3.9901±0.0610	4.2831±0.2163	4.4598±0.3019	4.4268±0.3117	4.4527±0.2643	4.3578±0.3057	4.1574±0.0617	4.6318±0.2665	4.4528±0.1450	4.6993±0.0678
014912-44-8	Tricyclo[4.4.0.02,7]dec-3-ene, 1,3-dimethyl-8-(1-methylethyl)-, stereoisomer	10.6582±0.3167	11.2534±0.3945	11.3398±0.4636	10.8727±0.5676	10.6735±0.3841	10.3835±0.3857	10.0439±0.1755	10.2299±0.5346	9.7582±0.2842	10.2933±0.3376

017066-67-0	beta-Selinene	20.4403±0.8835	23.8964±0.9259	23.7320±0.6901	23.1078±0.4368	24.0536±0.5699	24.0778±0.4905	23.2162±0.3713	25.5486±0.8131	24.8427±0.2829	25.7407±0.5436
005951-61-1	beta-Cadinene	3.1019±0.3049	3.2681±0.1280	3.3900±0.3890	3.2257±0.5107	3.4077±0.3104	2.8713±0.0371	2.9022±0.1575	3.2901±0.5171	3.0554±0.1944	3.4709±0.1457
000473-13-2	α-Selinene	27.3800±1.3756	31.1489±1.1643	30.7242±0.5759	29.6556±0.3623	30.2727±0.3392	30.2304±0.5889	29.4599±0.6439	31.0536±0.5904	30.2937±0.3930	30.8699±0.6808
029621-78-1	1-methyl-4-(1,2,2-trimethylcyclopentyl) cyclohexa-1,3-diene	12.1863±0.5832	12.7823±0.2978	12.6248±0.1621	11.8853±0.2893	11.3235±0.2294	11.0349±0.2211	10.9458±0.3184	10.1674±0.2429	9.8341±0.1971	9.7511±0.2753
010208-80-7	α-muurolene	18.6559±0.9852	21.3272±0.8438	21.0226±0.2668	20.2671±0.0494	20.7823±0.0666	20.9535±0.3954	20.3765±0.3678	21.4606±0.2818	20.9596±0.2312	21.4001±0.4813
016982-00-6	1-methyl-4-[(1R)-1,2,2-trimethylcyclop entyl] benzene	36.0759±2.1631	41.8515±1.0312	41.5115±0.5158	40.1694±0.1641	41.3179±0.4186	42.4209±0.7935	40.9186±0.7192	43.6759±0.4769	42.8874±0.5139	43.2515±0.9813
028400-12-6	alpha-alaskene	49.6728±2.5809	55.8611±1.9004	54.9135±0.6889	52.7628±0.1592	49.0728±7.7491	54.1795±1.1052	52.7865±1.0983	54.4342±0.4023	53.3066±0.7941	53.7170±1.2528
016204-67-4	1,1,4,5,6-Pentamethyl-2,3-dihydro-1H-indene	3.6781±0.2133	4.1547±0.1559	4.0379±0.0134	3.8552±0.0307	3.8967±0.0291	3.9057±0.0983	3.7983±0.1197	3.9468±0.0538	3.7891±0.0838	3.8687±0.1056
000483-76-1	(+)-delta-Cadinene	63.4476±3.2799	71.2019±2.4000	69.8715±0.8710	67.1972±0.2228	68.2028±0.2681	68.7953±1.4588	67.0950±1.5162	68.9530±0.5522	67.1424±0.9590	67.5881±1.4723
997220-96-6	gamma-Curcumene	6.8268±0.2930	6.7537±0.0406	6.8650±0.1317	2.1089±3.6527	3.7189±3.2261	1.8155±3.1445	3.8275±3.3306	0.0000±0.0000	0.0000±0.0000	1.5182±2.6295
053585-13-0	(4E)-1-methyl-4-(6-methylhept-5-en-2-ylidene) cyclohexene	9.4132±0.6830	10.5354±0.5297	10.1442±0.1972	13.9403±3.5361	12.0810±3.1228	13.8573±3.2122	11.6732±3.0417	14.9266±0.0719	14.5731±0.2564	12.9659±2.5563
024406-05-1	alpha-Cadinene	8.2840±0.4406	9.2934±0.3231	9.1199±0.1170	8.7470±0.0334	8.7997±0.0258	8.9545±0.1957	8.6389±0.2019	8.8231±0.0360	8.7755±0.1310	8.6434±0.1794
000515-17-3	gamma-selinene	4.5486±0.2626	5.1290±0.1714	5.0593±0.0759	4.8237±0.0504	4.8788±0.0171	4.9819±0.0974	4.7866±0.0982	4.9536±0.0512	4.9326±0.0659	4.8777±0.1007
021391-99-1	alpha-Calacorene	8.9060±0.4760	10.1146±0.3527	9.9173±0.1432	9.5467±0.0102	9.7062±0.0479	9.8174±0.2223	9.4841±0.1841	9.9072±0.0863	9.6793±0.1388	9.6769±0.2477
017627-44-0	alpha-bisabolene	4.3349±0.2181	4.8404±0.1556	4.7194±0.0815	4.5075±0.0301	4.5417±0.0145	4.5414±0.1144	4.4474±0.1027	4.5133±0.0359	4.4369±0.0589	4.4074±0.0642
021657-90-9	hedycaryol	6.6144±0.3534	7.4807±0.2571	7.2942±0.1090	7.1022±0.0427	7.1642±0.0295	7.2700±0.1806	7.0651±0.1410	7.3591±0.0566	7.2404±0.0938	7.2875±0.1426
158930-41-7	Eremophila ketone	2.9696±0.1537	3.4367±0.1096	3.3438±0.0320	3.2531±0.0160	3.2647±0.0138	3.3184±0.0693	3.2297±0.0559	3.3809±0.0319	3.3089±0.0539	3.3654±0.0773
020321-73-7	7-methoxy-2,2-dimethyl-3H-chromen-4-one	1.6582±1.4371	2.7512±0.1176	2.6733±0.0375	2.5895±0.0211	2.6511±0.1014	2.6254±0.0682	0.8399±1.4547	2.5547±0.0829	2.5884±0.0412	2.5379±0.0220
000077-53-2	(+)-Cedrol	3.3893±0.2461	3.9536±0.1807	3.8186±0.0392	3.9365±0.0329	3.9898±0.0236	3.7358±0.3132	3.7157±0.2576	4.1477±0.0677	3.9611±0.0356	4.1241±0.0837
002050-24-0	1,3-diethyl-5-methylbenzene	0.5045±0.4384	0.8080±0.0240	0.5284±0.4577	0.2588±0.4482	0.2569±0.4450	0.2545±0.4408	0.0000±0.0000	0.8108±0.0121	0.7724±0.0114	0.7723±0.0232
007786-67-6	5-Methyl-2-prop-1-en-2-yl-cyclohexan-1-ol	0.3477±0.3012	0.0000±0.0000	0.0000±0.0000	0.3509±0.3039	0.1704±0.2952	0.1686±0.2921	0.1829±0.3168	0.3442±0.2981	0.0000±0.0000	0.1668±0.2889
092471-23-3	(1S,2R,5R)-2-(2-Hydroxypropan-2-yl)-5-methylcyclohexanol	0.0000±0.0000	0.0000±0.0000	0.0000±0.0000	1.1538±0.1151	1.0509±0.0277	0.0000±0.0000	2.5462±0.0622	1.7144±0.0220	0.0000±0.0000	2.9333±0.3381
138663-70-4	2-(1-Hydroxy-1-methylethyl)-5-methyl cyclohexanol	0.0000±0.0000	0.0000±0.0000	0.0000±0.0000	0.0000±0.0000	0.0000±0.0000	0.0000±0.0000	1.0840±0.0512	0.0000±0.0000	0.0000±0.0000	1.1943±0.1341
000536-60-7	Cuminol	0.0000±0.0000	0.0000±0.0000	0.0000±0.0000	0.0000±0.0000	0.6791±0.0562	0.7796±0.0184	0.0000±0.0000	0.9424±0.0261	0.9889±0.0185	0.7667±0.0322

017699-14-8	(-)-Alpha-Cubebene	0.0000±0.0000	0.0000±0.0000	0.0000±0.0000	0.0000±0.0000	4.5182±7.8257	0.0000±0.0000	0.0000±0.0000	0.0000±0.0000	0.0000±0.0000	0.0000±0.0000
913176-41-7	4,8,11,11-tetramethylbicyclo [7.2.0] undec-3-en-5-ol	0.0000±0.0000	0.0000±0.0000	0.0000±0.0000	0.0000±0.0000	0.9102±1.5765	0.0000±0.0000	0.0000±0.0000	0.0000±0.0000	0.0000±0.0000	0.0000±0.0000
997332-93-7	2-Pentenoic acid, 3-methyl-5-(2,6,6-trimethyl-1-cyclohex enyl)	0.0000±0.0000	0.0000±0.0000	0.0000±0.0000	0.0000±0.0000	0.6756±1.1703	0.0000±0.0000	0.0000±0.0000	0.0000±0.0000	0.0000±0.0000	0.6981±1.2092
000481-34-5	alpha-cadinol	0.0000±0.0000	0.0000±0.0000	0.0000±0.0000	0.0000±0.0000	0.5635±0.9760	0.0000±0.0000	0.0000±0.0000	2.0686±0.2491	1.1511±0.9970	1.9685±0.2760
000502-47-6	Citronellic Acid	0.0000±0.0000	0.0000±0.0000	0.0000±0.0000	0.0000±0.0000	0.0000±0.0000	0.0000±0.0000	0.0000±0.0000	0.9139±0.0310	0.3278±0.5678	0.0000±0.0000
043219-80-3	(1R,4S,5S)-1,8-Dimethyl-4-(prop-1-en-2-yl) spiro [4.5] dec-7-ene	0.0000±0.0000	0.0000±0.0000	0.0000±0.0000	0.0000±0.0000	0.5297±0.9174	0.0000±0.0000	0.0000±0.0000	0.9103±0.9431	0.2682±0.4646	1.7351±0.1338

Table S2 Relative content of qualitative changed components detected in ATVO, physical mixture, and MAPE after 1, 3, and 5 d of exposure to intense light ($\bar{x}$±sd, n=3)

CAS	Compound name	Untreated ATVO	1d-M	1d-O	1d-P	3d-M	3d-O	3d-P	5d-M	5d-O	5d-P
007785-26-4	(1S)-(-)-alpha-Pinene	2.1473±0.1305	1.0637±0.2156	1.2388±0.2242	2.1743±0.0242	0.0000±0.0000	0.0000±0.0000	1.5709±0.1182	0.0000±0.0000	0.0000±0.0000	0.6827±0.1105
005794-04-7	Bicyclo[2.2.1]heptane	1.8177±0.1045	1.0677±0.1546	1.2313±0.1847	1.8902±0.0146	0.0000±0.0000	0.0000±0.0000	1.2976±0.0849	0.0000±0.0000	0.0000±0.0000	0.5807±0.1049
000127-91-3	Beta-Pinene	2.3273±0.1394	1.4715±0.1730	1.6591±0.2524	2.3675±0.0214	0.5904±0.0758	0.4356±0.3776	1.6582±0.1415	0.0000±0.0000	0.0000±0.0000	0.8106±0.0813
000500-00-5	p-Menth-3-Ene	0.8131±0.0442	0.3924±0.3423	0.6200±0.0708	0.8369±0.0106	0.0000±0.0000	0.0000±0.0000	0.6405±0.0408	0.0000±0.0000	0.0000±0.0000	0.0000±0.0000
000110-93-0	6-Methyl-5-hepten-2-one	0.7945±0.0499	0.7313±0.0304	0.7610±0.0434	0.7461±0.0012	0.4886±0.0291	0.1785±0.3091	0.1842±0.3190	0.0000±0.0000	0.0000±0.0000	0.0000±0.0000
000124-18-5	Decane	0.3606±0.3129	0.0000±0.0000	0.0000±0.0000	0.0000±0.0000	0.0000±0.0000	0.0000±0.0000	0.0000±0.0000	0.0000±0.0000	0.0000±0.0000	0.0000±0.0000
000099-83-2	α-Phellandrene	1.9377±0.1024	1.4460±0.1204	1.5423±0.1315	1.8412±0.0373	0.7100±0.0680	0.7113±0.0835	1.4183±0.0746	0.0000±0.0000	0.0000±0.0000	0.7774±0.0567
997220-96-6	gamma-Curcumene	6.8268±0.2930	6.7537±0.0406	6.8650±0.1317	2.1089±3.6527	3.7189±3.2261	1.8155±3.1445	3.8275±3.3306	0.0000±0.0000	0.0000±0.0000	1.5182±2.6295
007786-67-6	5-Methyl-2-prop-1-en-2-yl-cyclohexan-1-ol	0.3477±0.3012	0.0000±0.0000	0.0000±0.0000	0.3509±0.3039	0.1704±0.2952	0.1686±0.2921	0.1829±0.3168	0.3442±0.2981	0.0000±0.0000	0.1668±0.2889
000536-60-7	Cuminol	0.0000±0.0000	0.0000±0.0000	0.0000±0.0000	0.0000±0.0000	0.6791±0.0562	0.7796±0.0184	0.0000±0.0000	0.9424±0.0261	0.9889±0.0185	0.7667±0.0322
000481-34-5	alpha-cadinol	0.0000±0.0000	0.0000±0.0000	0.0000±0.0000	0.0000±0.0000	0.5635±0.9760	0.0000±0.0000	0.0000±0.0000	2.0686±0.2491	1.1511±0.9970	1.9685±0.2760
000502-47-6	Citronellic Acid	0.0000±0.0000	0.0000±0.0000	0.0000±0.0000	0.0000±0.0000	0.0000±0.0000	0.0000±0.0000	0.0000±0.0000	0.9139±0.0310	0.3278±0.5678	0.0000±0.0000
043219-80-3	(1R,4S,5S)-1,8-Dimethyl-4-(prop-1-en-2-yl)spiro[4.5]dec-7-ene	0.0000±0.0000	0.0000±0.0000	0.0000±0.0000	0.0000±0.0000	0.5297±0.9174	0.0000±0.0000	0.0000±0.0000	0.9103±0.9431	0.2682±0.4646	1.7351±0.1338

In vitro release studies

1.1 Preparation of release media

Artificial gastric fluid: Accurately transferred 16.4 mL dilute hydrochloric acid, diluted with water to approximately 800 mL, added 10 g pepsin, diluted with water to 1000 mL, to obtain artificial gastric fluid.

Artificial intestinal fluid: Accurately weighed 6.8 g potassium dihydrogen phosphate in 500 mL of water, dissolved, adjusted the pH to 6.8 with 0.1 mol/L sodium hydroxide solution; Then 10 g pancreatin was dissolved in a suitable amount of water, mixed the two solutions, diluted with water to 1000 mL to obtain the solution.

1.2 GC-MS conditions

Chromatographic conditions: HP-5 quartz capillary column (30 m×0.25 mm×0.25 μm) was used; Temperature program: the initial temperature was 50 °C, and increased to 140 °C at a rate of 15 °C/min, then rose to 144 °C at 0.4 °C/min and kept for 5 min, and then increased to 250 °C at 10 °C/min and kept for 2 min; Injection volume was 1 μL, and split ratio was 10:1. Solvent delay was 3 min, and carrier gas was high purity helium (99.999%).

Mass spectrometry conditions were as follows: Electron impact ion source, electron energy of 70 eV, ion source temperature of 230 °C, quadrupole temperature of 150 °C, and scan mode of Scan.

1.3 Equilibrium solubility of ATVO

An excess of ATVOs were taken in stoppered test tubes, 20 mL of artificial stomachic fluid and artificial intestinal fluid were added separately, then placed in a water bath shaker at 37 °C and oscillated for 24 hours at 100 r/min. 2 mL of saturated solution was pipetted precisely, added 2 mL n-hexane, vortexed for 3 min, then collected the upper layer and dehydrated with anhydrous sodium sulfate, filtered and determined by "1.2" finally. The equilibrium solubility of β-asarone and α-asarone of ATVO in artificial gastric fluid was 210.22 $\mu g \cdot mL^{-1}$ and 32.76 $\mu g \cdot mL^{-1}$, respectively; the equilibrium solubility of β-asarone and α-asarone in artificial intestinal fluid was 236.56 $\mu g \cdot mL^{-1}$ and 46.90 $\mu g \cdot mL^{-1}$, separately.

1.4 Preparation of reference solution

Accurately weighed appropriate amounts of β-asarone and α-asarone standard substances, diluted with n-hexane to obtain reference stock solutions with concentrations of 0.9800 mg/mL and 2.0854 mg/mL respectively.

1.5 Preparation of test solution

Appropriate amounts of ATVO and Pickering emulsion were pipetted into dialysis bags (with a molecular weight cut-off of 300 kDa), seperately. 200 mL of artificial gastric fluid and artificial intestinal fluid were release medium, and the temperature of shaker was set at 37 °C with a rotational speed of 100 rpm. When temperature rose to set value, the dialysis bags containing ATVO or Pickering emulsion were put into a beaker, and started immediately and timed. At 48 h of dissolution, 2 mL of dissolution solution was taken, 2 mL of n-hexane was added, vortexed for 3 min, and left to stand, the upper solution was collected, dehydrated with anhydrous sodium sulfate, and passed through 0.22 μm microporous filtration membrane, and the filtrate of renewed filtration solution was taken as the test solution.

1.6 Method validation of the in vitro solubility study

1.6.1 Specificity

Appropriate amounts of blank solvent, mixed control solution and test solution were taken and determined according to conditions under "1.2". Figure S2 shows that at the retention time of β-asarone and α-asarone, there was no interference from the blank solvent, indicating good specificity of this method.

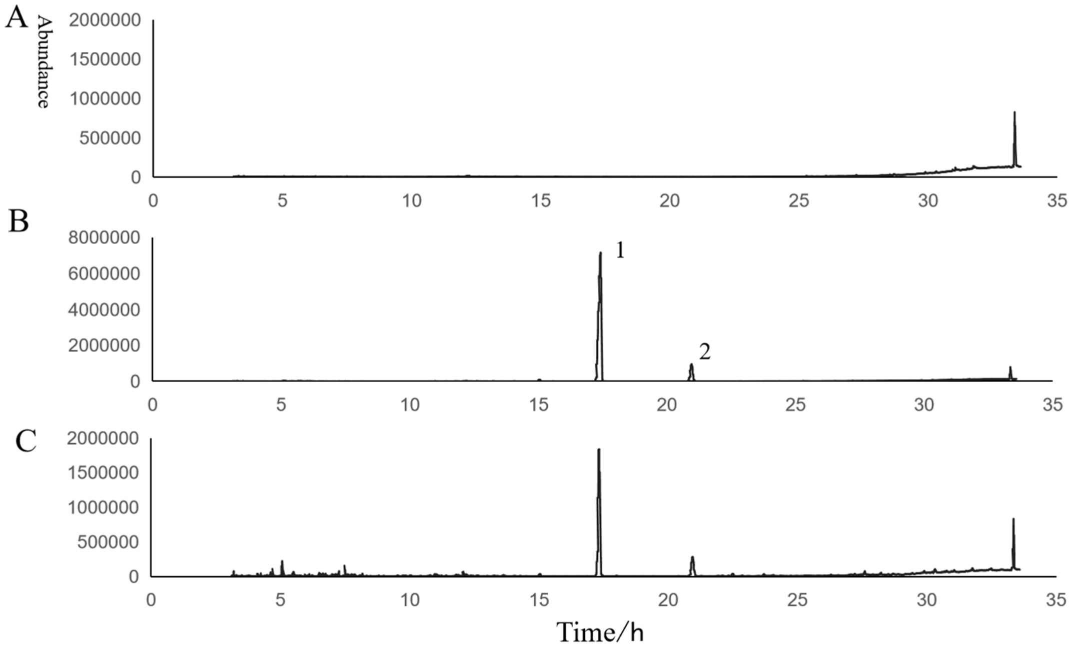

Figure S2 GC-MS chromatogram of β-asarone and α-asarone. (A) n-hexane; (B) β-asarone and α-asarone control solution; (C) sample at 48 h of release of ATVO from artificial intestinal fluid; 1 β-asarone; 2 α-asarone

1.6.2 Standard curve

Pipette appropriate amount of *β*-asarone stock solution into 2 mL volumetric flask, fixed to acquire the standard solution of *β*-asarone, diluted 2, 4, 8, 20, 50, 100, 200, 400 times with n-hexane, respectively; appropriate amount of *α*-asarone stock solution in a 2 mL volumetric flask, fixed to obtain standard solution of *α*-asarone, and diluted 2, 4, 8, 20, 50 times, respectively. Then measured according to conditions under "1.2", and the regression equation was calculated by plotting the standard curve with the peak area of components (*Y*) and the concentration of standard solution (*X*, $\mu g\cdot mL^{-1}$). The results are shown in Table S3, and both *β*-asarone and *α*-asarone showed good linearity within linear range.

Table S3 Standard curves of *β*-asarone and *α*-asarone

Components	Regression equation	*r*	Linear range（μg/mL）
β-asarone	$Y = 1226016.20X - 102648.39$	0.9999	0.7350 - 294.0000
α-asarone	$Y = 1278279.87X - 315321.30$	0.9991	1.0427 - 52.1360

1.6.3 Precision

Precisely transferred appropriate amounts of *β*-asarone and *α*-asarone stock solutions into 2 mL volumetric flasks, fixed and filtered, and repeatted sample injection 6 times referring "1.2 GC-MS Conditions". The RSD of peak areas for *β*-asarone and *α*-asarone were 0.84% and 0.93% respectively, both less than 3.00%, indicating good precision of the instrument.

Table S4 Precision of *β*-asarone and *α*-asarone

Sample	*β*-asarone	*α*-asarone
1	529504665	66589626
2	531444952	67039803
3	534757195	67468182
4	536090847	67582475
5	538944249	68030117
6	541499583	68293428
RSD%	0.84	0.93

1.6.4 Reproducibility

Six portions of the dissolution solution of ATVO in artificial intestinal fluid at 48 h were pipetted precisely, prepared according to the method under "1.5", and then determined according to "1.2". The results showed that the RSDs of the peak areas of *β*-asarone and *α*-asarone were 2.99% and 3.06% respectively, indicating good reproducibility.

Table S5 Reproducibility of β-asarone and α-asarone

Sample	β-asarone	α-asarone
1	90333951	16694391
2	91829482	17570772
3	91165596	16943015
4	93167610	17551038
5	94843862	17825408
6	95127661	17981504
RSD%	2.99	3.06

1.6.5 Stability

Precisely pipetted the dissolution solution of ATVO in artificial intestinal fluid at 48 h, then processed according to the method under "1.4", and determined according to "1.2" at 0, 2, 4, 8, 12, 24 h, respectively. The RSD of the peak areas of β-asarone and α-asarone were 2.40% and 2.65% respectively, indicating that the test sample was stable within 24 h.

Table S6 Stability of β-asarone and α-asarone

Time/h	β-asarone	α-asarone
0	99763504	19078269
2	101386351	19230327
4	100565233	19166145
8	101789968	19159452
12	102978219	19568972
24	106665695	20431836
RSD%	2.40	2.65

1.6.6 Recovery

Six portions of the release medium of ATVO at 48 hours were took, controls were added at 100% according to the content of β-asarone and α-asarone in the dissolved solution of ATVO at 48 h, respectively. Then, the text solutions were prepared and analyzed. RSDs of recoveries were 2.35% and 2.42% for β-asarone and α-asarone in ATVO, respectively, indicating good recovery rates.

Table S7 Recoveries of β-asarone and α-asarone (n =6)

Components	Content in samples（μg/mL）	Amount of controls added（μg/mL）	Measured value（μg/mL）	Recovery rate%	RSD %
β-asarone	76.08	75.73	150.14	97.52	2.35
	74.44	75.73	149.15	98.36	
	77.67	75.73	149.88	95.07	
	77.44	75.73	149.77	95.23	
	74.98	75.73	149.75	98.44	

	73.76	75.73	150.63	101.20	
	13.31	13.88	27.66	103.92	
	13.99	13.88	27.66	98.95	
α-asarone	13.50	13.88	27.50	101.34	2.42
	13.98	13.88	27.65	98.94	
	14.19	13.88	27.70	97.77	
	14.31	13.88	27.82	97.74	

In vivo pharmacokinetic studies

2.1 Methodological investigations

2.1.1 Specificity

After processing blank plasma, blank plasma spiked with control samples, and post-dose plasma according to plasma sample processing method, they were analyzed by GC-MS according to "1.2". As shown in Figure S3, endogenous substances in the plasma did not interfere with *β*-asarone and *α*-asarone, indicating good method specificity.

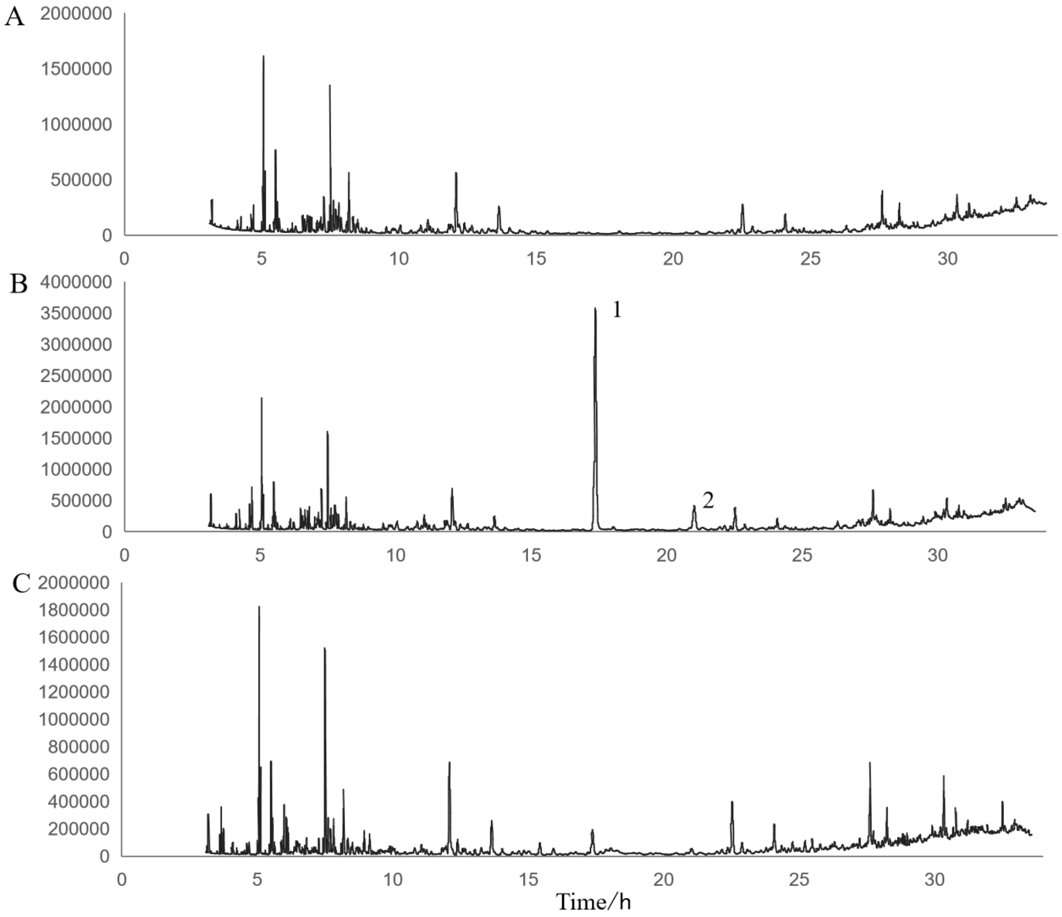

Figure S3 Chromatogram of plasma sample. A blank plasma; B blank plasma spiked with control samples; C post-dose plasma. 1 *β*-asarone; 2 *α*-asarone

2.1.2 Standard curve

150 µL of blank plasma was accurately pipetted and mixed with a series of standard solutions to prepare samples with *β*-asarone blood drug concentrations of 147, 73.5, 36.75, 18.375, 7.35, 3.675, and 1.47 µg/mL, and *α*-asarone concentrations of 26.068, 13.034, 6.517, 3.2585, 1.3034, and 0.6517 µg/mL. The samples were processed according to the plasma sample processing method and analyzed by

GC-MS according to "1.2". A standard curve was plotted with the concentration of the analyte in plasma as the x-axis (X, μg/mL) and the peak area of the analyte as the y-axis. The results in Table S8 show that the correlation coefficients (r) were all greater than 0.999, indicating a good linear relationship for *β*-asarone and *α*-asarone within their respective ranges.

Table S8 Standard curves of *β*-asarone and *α*-asarone in plasma

Components	Regression equation	*r*	Linear range（μg/mL）
β-asarone	*Y*=1391143.64*X*-3995249.20	0.9995	1.47 ~ 147
α-asarone	*Y*=1017826.15*X*-907595.45	0.9993	0.6517 ~ 26.068

2.1.3 Precision

Low, medium, and high-quality control (QC) samples were prepared at three concentration levels for both *β*-asarone (5.88, 29.40, 102.90 μg/mL) and *α*-asarone (1.04272, 5.2136, 18.2476 μg/mL). Six samples in parallel were detected for each concentration within a 24-hour period to determine intra-day precision. Over a period of 3 days, samples were continuously analyzed to calculate inter-day precision. The results presented in Table S9 demonstrate that the intra-day and inter-day precision (RSD) of *β*-asarone and *α*-asarone at the three different concentration levels in the QC samples met the required criteria.

Table S9 Intra-day and inter-day precision of *β*-asarone and *α*-asarone in rat plasma (*n*=6).

Components	Concentration（μg/mL）	Intra-day RSD (%)	Inter-day RSD (%)
β-asarone	5.88	5.54	8.02
	29.40	5.29	7.85
	102.90	9.29	7.41
α-asarone	1.04272	11.26	11.70
	5.2136	5.30	7.21
	18.2476	10.42	8.02

2.1.4 Extraction recovery and matrix effects

150 μL of blank plasma was precisely pipetted and rejoin low, medium, and high concentrations of QC samples, seperately. Executed sample processing according to plasma sample processing method. Six replicates for each concentration and recorded the resulting peak areas as A. Additionally, six replicates of blank plasma were prepared in accordance with plasma sample processing method and then added an equal amount of QC samples. Upon analysis, document the peak areas as B. Subsequently, QC samples at low, medium, and high concentrations were processed in

alignment with plasma sample processing method, analyzed and designated their peak areas as C. The extraction recovery was the ratio of A to B, and the matrix effect was the ratio of B to C. The outcomes outlined in Table S10 authentically demonstrate that the extraction recoveries and matrix effects of *β*-asarone and *α*-asarone consistently fall within the range of 90.00% to 110.00%.

Table S10 Extraction recovery and matrix effects of *β*-asarone and *α*-asarone in rat plasma (*n* = 6).

Components	Concentration (μg/mL)	Extraction recovery (%)	RSD (%)	Matrix effects (%)	RSD (%)
β-asarone	5.88	101.51	6.33	103.39	6.35
	29.40	93.76	4.34	101.81	4.85
	102.90	92.76	2.73	105.67	4.94
α-asarone	1.04272	102.21	7.86	95.97	9.16
	5.2136	92.89	5.15	106.79	4.26
	18.2476	94.82	3.61	108.21	3.28

2.1.5 Stability

QC samples at low, medium, and high concentrations were subjected to room temperature stability (24 hours at room temperature) and freeze-thaw stability (three cycles of freeze-thaw). Six replicates were prepared for each concentration, and the results are presented in Table S11. The RSDs of short-term and freeze-thaw stability were less than 8.72% and 5.82% for *β*-asarone, 8.88% and 7.39% for *α*-asarone, respectively.

Table S11 Stability of *β*-asarone and *α*-asarone in rat plasma（*n* = 6）

Components	Concentration (μg/mL)	Short-term stability RSD (%)	Freeze-thaw stability RSD (%)
β-asarone	5.88	6.10	3.71
	29.40	8.72	5.71
	102.90	2.02	5.82
α-asarone	1.04272	4.69	5.54
	5.2136	8.88	6.46
	18.2476	2.47	7.39

2.2 Blood-drug concentration of *β*-asarone and *α*-asarone after oral administration of ATVO and MAPE formulation

Table S12 Blood-drug concentration of *β*-asarone and *α*-asarone in MAPE and ATVO ($n = 6$, $\bar{x}$±sd)

Time (h)	*β*-asarone		*α*-asarone	
	ATVO (mg/L)	MAPE (mg/L)	ATVO (mg/L)	MAPE (mg/L)
0.08	3.99±4.95	8.75±1.01	2.30±4.36	4.43±2.16
0.25	7.43±4.40	10.47±2.64	3.16±2.11	4.35±0.85
0.5	7.70±2.02	8.62±1.56	2.84±1.58	3.56±0.67
0.75	8.85±2.28	14.09±5.53	3.86±1.19	6.83±3.13
1	8.24±2.58	8.73±2.75	4.01±1.41	3.51±1.41
1.5	9.88±3.02	9.00±3.46	4.22±1.01	3.96±2.07
2	11.79±6.35	8.00±2.16	5.08±3.05	2.63±1.49
3	8.39±0.77	10.70±4.38	3.25±0.68	4.17±2.27
4	8.79±6.66	8.17±1.73	3.53±3.46	3.06±0.68
6	8.97±3.31	9.62±3.27	3.47±2.06	4.95±2.18
8	8.34±5.18	6.94±2.92	3.22±3.29	2.62±1.57
12	6.31±1.53	10.04±7.92	3.33±2.11	6.66±4.80
